# P-386. Assessing Implementation of Evidence-Based Practice Recommendations for Prevention of Surgical Site Infections Through a Statewide Survey of Nebraska Hospitals

**DOI:** 10.1093/ofid/ofae631.587

**Published:** 2025-01-29

**Authors:** Lacey Pavlovsky, Fnu Kanishka, Jenna Preusker, Mikayla Baker, Tammy Hill, Laura A Hoogestraat, Mary W Kinyoun, Emily Nelson, Rebecca Martinez, Robert G Penn, Angel Plueger, Jody Scebold, Kate Tyner, Trevor C Van Schooneveld, Renuga Vivekanandan, Muhammad Salman Ashraf

**Affiliations:** Nebraska Department of Health and Human Services, Bellevue, Nebraska; Nebraska Department of Health and Human Services, University of Nebraska-Lincoln, Lincoln, Nebraska; Nebraska Medicine/Nebraska DHHS, Norfolk, Nebraska; Nebraska Methodist Health System, Omaha, Nebraska; Methodist Women's Hospital, Omaha, Nebraska; Faith Regional Health Services, Norfolk, Nebraska; UNMC/Nebraska Medicine, Omaha, Nebraska; Nebraska Medicine, Omaha, Nebraska; Nebraska Infection Control Assessment and Promotion Program, Nebraska Medicine, Omaha Nebraska, Omaha, Nebraska; Infectious Diseases Associates, PC, Omaha, Nebraska; CHI Health Creighton University Medical Center-Bergan Mercy, Omaha, Nebraska; Nebraska Medicine, Omaha, Nebraska; Nebraska Medicine, Omaha, Nebraska; University of Nebraska Medical Center, Omaha, NE; Creighton University Medical Center CHI Health, omaha, Nebraska; University of Nebraska Medical Center and Nebraska DHHS, Omaha, Nebraska

## Abstract

**Background:**

Almost half of surgical site infections (SSIs) are preventable by application of evidence-based strategies. An online survey of Nebraska hospitals was conducted by the Nebraska Healthcare-Associated Infections/Antimicrobial Resistance Advisory Council’s SSI Subcommittee between March and May 2023 to assess implementation of evidence-based practice recommendations (EBPR) from national guidelines.

**Figure:**
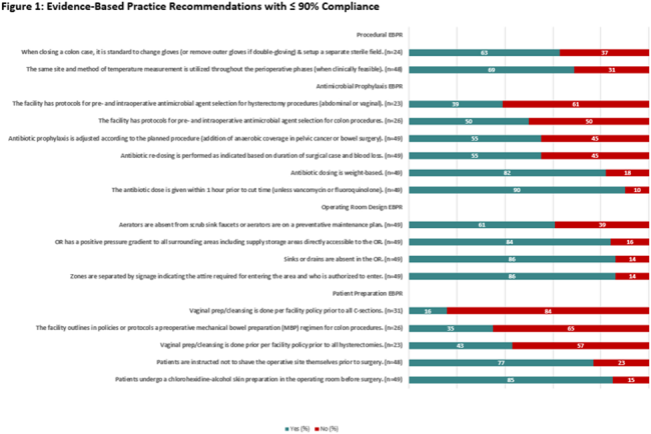

**Methods:**

Infection preventionists completed the survey in collaboration with their perioperative teams. In addition to facility demographics, data was collected on general SSI prevention practices along with those specific to colon procedures, hysterectomies, and cesarean sections. Implementation frequencies were calculated for each EBPR after excluding missing responses.

**Figure 2:**
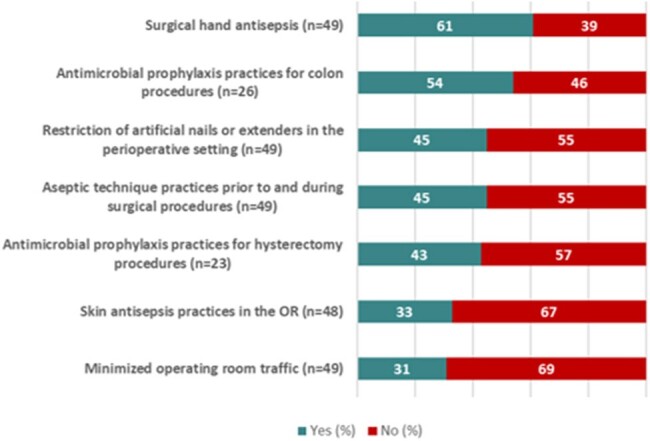
Routine Audits Conducted by Facilities

**Results:**

Response rates for acute care and critical access hospitals were 72% (21/29) and 47% (30/64), respectively. Most EBPR (19/30) assessed were reported to be implemented by majority ( >50%) of the hospitals. The least implemented EBPR was vaginal cleansing prior to all cesarean sections (16%, 5/31) (Figure 1). Audits to monitor operating room traffic and skin antisepsis were conducted by approximately a third of hospitals (31%, 15/49 and 33%, 16/48, respectively) (Figure 2). Among hospitals that performed hysterectomies (n=23), less than half reported performing vaginal cleansing prior to all hysterectomies (43%), presence of policies/protocols for pre- and intraoperative antibiotic use for hysterectomies (39%) and monitoring of antimicrobial prophylaxis practices for hysterectomies (43%). Least implemented colon procedure specific EBPR in hospitals performing these procedures (n=26) included having standardized policies/procedures for mechanical bowel preparation (35%), presence of policies/protocols for pre- and intraoperative antibiotic use for colon procedures (50%,) and monitoring of antimicrobial prophylaxis practices for colon procedures (54%).

**Conclusion:**

Several EBPR for SSI prevention are not routinely implemented highlighting the need for educational efforts to raise awareness among hospitals. Future studies should focus on identifying practice barriers and mitigation strategies to increase implementation.

**Disclosures:**

**Trevor C. Van Schooneveld, MD, FSHEA, FIDSA**, AN2 Therapeutics: Grant/Research Support|BioMerieux: Grant/Research Support|BioMerieux: Honoraria|Thermo-Fisher: Honoraria **Renuga Vivekanandan, MD**, MDconsult: Ownership Interest **Muhammad Salman Ashraf, MBBS**, Merck & Co. Inc: Grant/Research Support

